# The differential polarizability of CHO cells can be used to monitor changes in metabolism

**DOI:** 10.1186/1753-6561-9-S9-P47

**Published:** 2015-12-14

**Authors:** Katrin Braasch, Bahareh S Rizi, Elham Salimi, Gregory Bridges, Douglas Thomson, Michael Butler

**Affiliations:** 1 Microbiology, University of Manitoba, Winnipeg, Manitoba, R3T 2N2, Canada; 2 Computer and Electrical Engineering, University of Manitoba, Winnipeg, Manitoba, R3T 2N2, Canada

## Background

A healthy cell has a well-balanced ionic content. Changes in the ionic content, flux, membrane and mitochondrial capacitance within the cells have been correlated to physiological and metabolic changes linked to early stages of apoptosis [1]. The ionic content and structure of individual cells allow them to be polarized in an applied electrical field with an alternating current in the radiofrequency range [2]. Any changes in the ionic content will then lead to changes in the response of the cells to the applied electric field. Hence, methods based on dielectric measurements are particularly promising for identifying early emerging subpopulations of apoptotic cells.

We have developed a prototype cytometer, which measures the dielectrophoretic properties of individual cells. This tracks the trajectory of individual cells as they pass through a bank of electrodes designed to differentially perturb the cells according to their polarizability. Cells are analyzed in the instrument (DEP cytometer) by their displacement in an electrical field with sensitivity of 0.1 µm and at a rate of 5 cells per second [3].

Bulk average capacitance measurements of a bioreactor cell population have been made possible by commercially available sterilizable probes. However, the analysis of single cells allows a unique insight into the metabolism and energy flow within cells. By using single cell analysis discoveries can be made that might otherwise be masked by the overall response of a cell population.

## Material and methods

Apoptosis was artificially induced using a starvation medium (no glucose or glutamine) and oligomycin to analyze the changing dielectric properties in CHO cells during cell demise. The onset and progression of apoptosis in cells was measured using a bulk capacitance probe, the DEP cytometer, trypan blue exclusion, and several fluorescent assays. All measurements were compared to a healthy control culture.

Cell samples taken from cultures were analyzed by the DEP cytometer with a sample size of around 600 cells. The changing polarizability of the cells was measured by a force index (FI), which was related to the electrical displacement of the cells. Analysis with the capacitance probe was done by running frequency sweeps in each treatment for 30 min at each sampling point to produce a β-dispersion plot.

## Results

A bulk, a single cell dielectric measurement, trypan blue exclusion and several fluorescent assays were used to monitor and compare the onset and progression of apoptosis in a starvation and oligomycin induced culture to a control culture.

**Figure 1:**
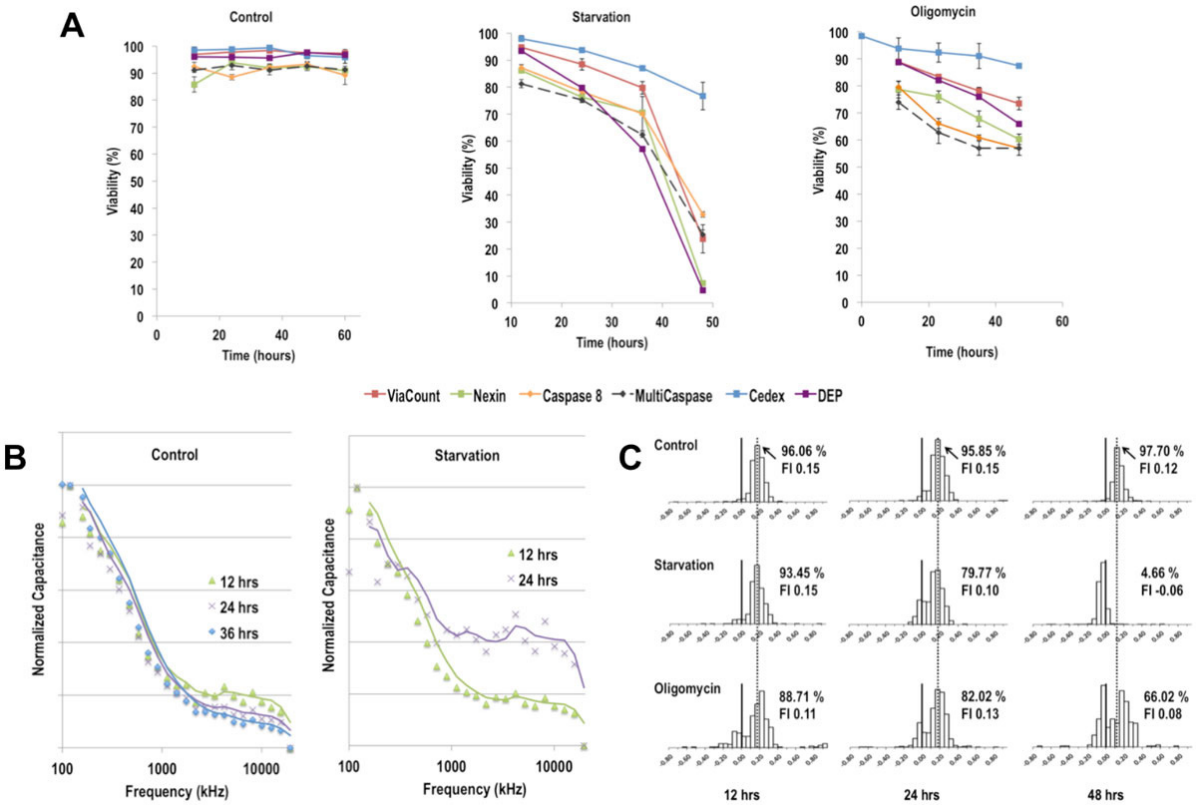
**Time-series measurements using (a) trypan blue, DEP, and several fluorescent apoptosis assays, (b) a capacitance probe, and (c) the DEP cytometer to detect the onset and progression of apoptosis during apoptosis induction**. The normalized capacitance vs. frequency was plotted for the capacitance probe. For the DEP cytometer the Force index (FI) distribution, DEP viability and average FI is displayed.

Figure 1A shows the different viabilities determined for all three cultures. While the changes in the starvation culture capacitance reading related with the ViaCount assay (not shown), the change in the viability identified with the DEP cytometer correlated with the AnnexinV assay. During starvation the lack of glucose led to a decline of ATP in the cells. The lack of ATP results in the inability of the cells to maintain their well-balanced ionic content. The subsequent change in the polarizabiltiy of the cells was picked up by the DEP cytometer. At the same time the number of phosphatidyl serine molecules on the outer leaflet of the cell membrane increased, which is detected by the Annexin V assay.

Oligomycin hinders the mitochondrial ATP production by inhibiting the ATP-Synthase. This leads to a change in the mitochondrial potential and eventually to the initiation of the caspase cascade. Hence, the lowest viability was determined using the caspase assays. As apoptosis progresses changes were also observed in the cytoplasm conductivity and cell membrane capacitance, which were detected using the DEP cytometer.

Figure 1B shows the capacitance data of the sampling points in a control and starvation culture as normalized capacitance versus frequency. In the control culture this resulting β-dispersion plot remained fairly consistent over time indicating that the overall dielectric properties of the cells in the culture did not change or varied significantly. In the starvation the initial 12 h measurement was very similar to the control culture. However, by 24 h the measurement showed an increased capacitance reading at higher frequencies. This change indicated the presence of a subpopulation of cells for which the dielectric properties changed due to the starvation treatment. The DEP cytometer results for the same cultures and an oligomycin treatment are shown in Figure 1C. For the DEP cytometer measurements the force index was calculated for each cell and their overall distribution was graphed. This distribution paints a clear picture of the overall heterogeneity in every culture - even the control. In the apoptosis induced cultures a small subpopulation with a decreased force index (compared to the control) was identified and quantified. During the progression of apoptosis in those cultures the force index continued to gradually decrease and a more prominent sub-population established itself.

## Conclusions

Changes in the dielectric properties of CHO cells can be used to monitor changes in cell health and metabolism during cell culture processes. These changes can be monitored using either an online bulk capacitance probe or a single cell analysis DEP cytometer prototype. While both methods detect emerging subpopulations single cell analysis allows for more distinct and sensitive monitoring of changes and rare events in a label-free, non-invasive and all electronic manner. In the DEP cytometer 600 cells can be analyzed in 10 min, which is half the time needed for the Annexin V assay with only 6% of the number of cells required.

## Acknowledgements

The authors would like to thank Aber Instruments Co. (Aberystwyth, UK) and EMD Millipore (Danvers, MA) for the use of their instruments for this study. We also thank the Natural Science and Engineering Research Council (NSERC) of Canada for funding through MabNet and the Faculty of Science (University of Manitoba) for supporting this work.
